# AI models based on gadoxetic acid–enhanced MRI to predict treatment response and prognosis after TACE in hepatocellular carcinoma

**DOI:** 10.3389/fonc.2026.1738531

**Published:** 2026-04-28

**Authors:** Jiangqin Ma, Linghong Kong, Wenlong Song, Xin Li, Xiaojing He, Xi Liu, Dajing Guo, Yangyang Liu

**Affiliations:** 1 Department of Radiology, Second Affiliated Hospital, Chongqing Medical University, Chongqing, China; 2 Department of Radiology, Beibei Affiliated Hospital of Chongqing Medical University, the Ninth People’s Hospital of Chongqing, Chongqing, China

**Keywords:** deep learning, Gd-EOB-DTPA, hepatocellular carcinoma, magnetic resonance imaging, radiomics, transarterial chemoembolization

## Abstract

**Objectives:**

To develop and validate radiomics and deep learning models based on the hepatobiliary phase (HBP) of gadoxetic acid-enhanced MRI (EOB-MRI) for the noninvasive prediction of treatment response and prognosis following transarterial chemoembolization (TACE) in hepatocellular carcinoma (HCC).

**Materials and methods:**

From April 2018 to September 2024, 160 patients with Barcelona Clinic Liver Cancer (BCLC) stage A or B HCC (>3 cm) were retrospectively enrolled and randomly divided into training (n = 112) and test (n = 48) sets. An independent cohort of 38 HCC patients was used for external validation. Twenty-six radiomic features were extracted using LASSO to construct a machine learning model using eXtreme gradient boosting (XGBoost). A deep convolutional neural network (DCNN) based on the ResNet50 architecture was used to develop a deep learning model. A clinical model was built via multivariate logistic regression. Model performance was evaluated by the area under the curve (AUC), calibration curves, and decision curve analysis (DCA). Kaplan–Meier analysis of combined deep learning and radiomics(DLR) scores was used to estimate overall survival in the follow-up cohort (n = 117).

**Results:**

BCLC stage (P = 0.035), tumor number (P = 0.015), and tumor size (P = 0.013) were independent clinical predictors. In the training set, the AUCs (95% CI) for the clinical, radiomics (XGBoost), and deep learning (DCNN) models were 0.79, 0.84, and 0.96, respectively. In the test set, the AUCs were 0.70, 0.80, and 0.92, respectively. In the external validation set, the AUCs were 0.77, 0.80, and 0.86, respectively. The DCNN model showed superior calibration and the highest net clinical benefit in DCA. In addition, multivariable Cox regression revealed that DLR model output was an independent risk factor for the overall survival (hazard ratio: 15.9, 95% CI: 4.49-56.33; p < 0.001).

**Conclusion:**

HBP-based AI models effectively predicted TACE response and prognosis in HCC patients, with the DCNN model showing the best performance. The integrated DLR model demonstrated high predictive accuracy and may serve as a reliable tool for individualized treatment planning in precision oncology.

## Introduction

Primary liver cancer poses a significant global health burden and is the sixth most commonly diagnosed cancer and the third leading cause of cancer-related death (1). The 5-year survival rate ranges from 5% to 30% (2). Approximately 80% of primary liver cancers are hepatocellular carcinoma (HCC) (3). Surgical resection and liver transplantation are the first-line recommended treatments for curing HCC, with 5-year survival rates above 60%. However, HCC often develops without symptoms and progresses rapidly, and more than half of patients are diagnosed at intermediate or advanced stages, making them ineligible for curative treatments (4). According to the Barcelona Clinic Liver Cancer (BCLC) guidelines, transarterial chemoembolization (TACE) is recommended as the first-line treatment for unresectable HCC, either alone or in combination with other therapies such as ablation, targeted therapy or immunotherapy. TACE achieves an objective response rate of approximately 52.5% and can effectively control tumor progression and prolong survival (5, 6). Additionally, it is commonly used as a bridging or downstaging strategy to reduce the tumor burden and help patients become eligible for surgical resection or liver transplantation. Nevertheless, the treatment response to TACE remains highly variable due to tumor heterogeneity, and nearly half of patients fail to achieve objective remission (5). Early identification of nonresponders is essential, as timely initiation of targeted therapy or immunotherapy has been shown to improve clinical outcomes (7–10).

Current predictive models integrate clinical variables (BCLC stage and subclassification, tumor burden, elevated serum alpha-fetoprotein (AFP), etc.) and morphological features (central location, bilobar distribution, segment I/IV involvement) (11–13). With advances in artificial intelligence (AI), radiomics and deep learning have enabled the extraction and analysis of high-dimensional data from medical images, improving disease diagnosis, prediction of treatment response, and assessment of prognosis. Radiomics involves the quantification of large sets of imaging features, whereas deep learning employs neural networks to capture complex spatial patterns within image data. Recent studies have reported pooled AUCs of 0.82–0.89 for machine learning models in predicting TACE response, highlighting their promising clinical potential (14–25). However, most previous studies have focused on extracellular MR contrast agents, with only a limited number of studies exploring gadoxetic acid (Gd-EOB-DTPA)-enhanced magnetic resonance imaging (EOB-MRI) (11, 26).

The hepatobiliary phase (HBP) of EOB-MRI provides functional insights into tumor pathology and the microenvironment, both of which are closely associated with treatment response. Previous research has demonstrated its superiority in predicting tumor differentiation, microvascular invasion (MVI) and invasiveness, all of which are critical for predicting treatment outcome and prognosis (27–30). Despite its clinical value, the application of AI to EOB-MRI remains limited, with studies focused primarily on small HCCs and conducted in small cohorts (26). The potential of EOB-MRI for predicting TACE response in larger tumors or those at BCLC B stage has yet to be thoroughly explored.

The aim of this study is to develop and validate radiomics and deep learning models based on HBP sequences from EOB-MRI for the noninvasive prediction of TACE response in patients with HCC. In addition, the prognostic performance of the deep learning and radiomics (DLR) models were further evaluated. This approach may facilitate the identification of patients most likely to benefit from TACE and support personalized treatment planning.

## Materials and methods

### Patients and MRI protocol

**Figure 1:**
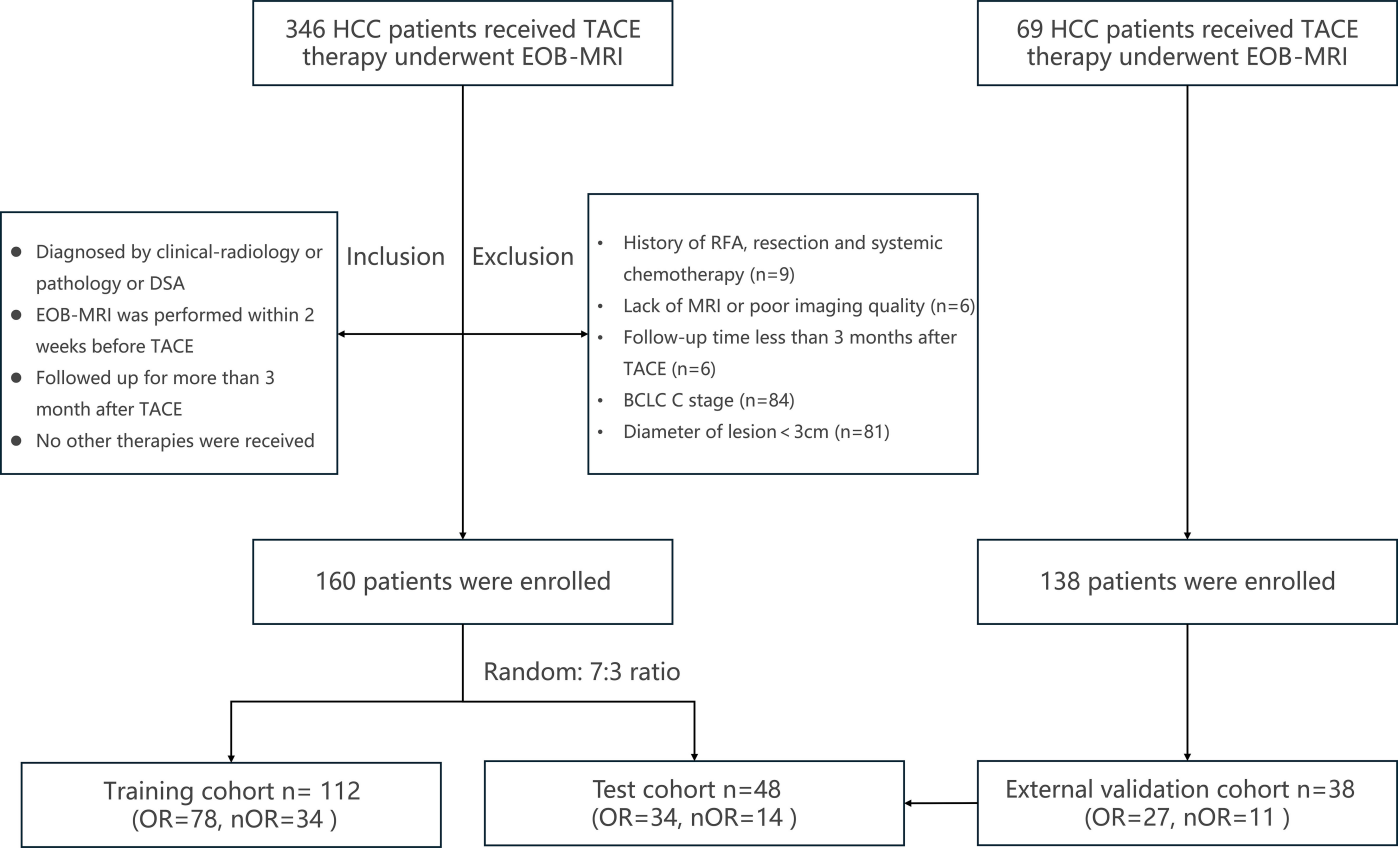
Flowchart of patient enrolment and study design. HCC, hepatocellular carcinoma; TACE, transcatheter arterial chemoembolization; EOB-MRI, gadoxetate disodium-enhanced magnetic resonance imaging; DSA, digital subtraction angiography; RFA, radiofrequency ablation; BCLC, Barcelona Clinic Liver Cancer; OR, objective response; nOR, non-objective response.

This study was approved by the hospital’s Ethics Committee (Approval No. [2022]788). The data were obtained from a prospective project, and this work represents a retrospective analysis of those data. Therefore, the requirement for additional informed consent was waived. A total of 346 consecutive patients who were diagnosed with BCLC stage A or B HCC; confirmed by pathology, clinical-radiologic criteria, or digital subtraction angiography; and treated with TACE between April 2018 and September 2024 were initially enrolled. The inclusion criteria were as follows: (1) confirmed diagnosis of HCC; (2) underwent EOB-MRI within 2 weeks prior to TACE; (3) complete clinical data, including demographic information, hepatitis status, AFP levels, and liver function tests; and (4) follow-up of at least 3 months after TACE. The exclusion criteria were as follows: (1) underwent other treatments, such as radiofrequency ablation, surgical resection, radiotherapy, or systemic chemotherapy; (2) poor image quality; (3) the presence of other malignancies; (4) a diagnosis of advanced HCC (BCLC stage C); and (5) a tumor diameter less than 3 cm. A total of 160 HCC patients were ultimately included. The patient recruitment process is illustrated in **Figure 1**. External validation was performed on 38 HCC patients treated with TACE in another branch of the institution (The second affiliated hospital of Chongqing Medical University, Yuzhong branch). The inclusion and exclusion criteria for these patients were consistent with those of the previous cohort.

MRI examinations were performed on a 3.0T (Magnetom Prisma, Siemens Healthineers, Erlangen, Germany) or 1.5T (Signa HDxt, Milwaukee, WI, USA) scanner with an 18-channel body phased array coil. All patients underwent upper abdominal EOB-MRI. Gd-EOB-DTPA (Primovist, Bayer Schering Pharma, Berlin, Germany) was administered intravenously at a dose of 0.025 mmol/kg at a rate of 1.0 mL/s, followed by a 20 mL saline flush at the same rate. The detailed scanning parameters are described in **Supplementary Table 1**.

### TACE treatment and reference standards for TACE response

All enrolled patients received TACE treatment, including conventional TACE (cTACE), drug-eluting bead TACE (DEB-TACE) and combined TACE. The choice between cTACE and DEB-TACE was determined on the basis of liver function and tumor characteristics. With the exception of embolic agents, the basic treatment processes of cTACE and DEB-TACE are similar. cTACE uses Lipiodol (Yantai, China), gelatin sponge particles, and polyvinyl alcohol as embolic agents. For DEB-TACE, CalliSpheres^®^ microspheres (Merit Medical, USA) with a diameter of 100–300 μm or 300–500 μm were used as carriers and were loaded with 60–80 mg of epirubicin or pirarubicin. All procedures were performed by interventional radiologists with over 10 years of clinical experience who adhered to current practice guidelines. Following TACE, all patients were admitted for postoperative supportive care and routine management.

**Figure 2:**
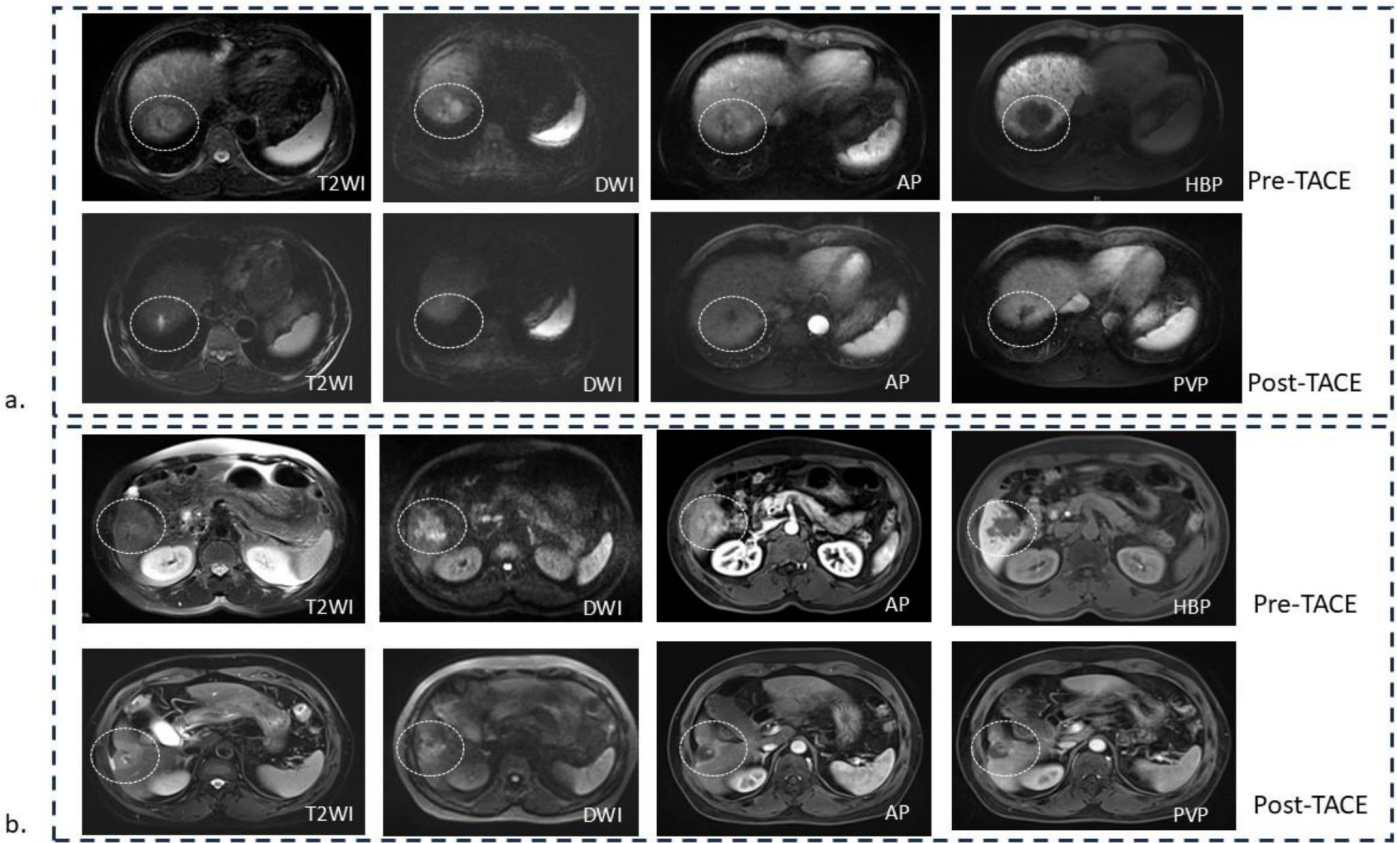
Representative examples of post-TACE response in HCC patients. **(a)** A 57-year-old male patient presented with a 43 mm mass in the right hepatic lobe, showing slightly hyperintense on T2WI and DWI, marked arterial enhancement, and hypointensity on the HBP. After TACE, the lesion significantly reduced in size with no residual enhancement, corresponding to OR according to mRECIST criteria. **(b)** A 51-year-old male patient showed a 38 mm lobulated mass with irregular margins in the right hepatic lobe, hyperintense on T2WI and DWI, exhibiting marked arterial enhancement and hypointensity on HBP. After TACE, the lesion exhibited partial necrosis, but the reduction in tumor size was less than 30%, consistent with SD. TACE, transcatheter arterial chemoembolization; HCC, hepatocellular carcinoma; T2WI, T2-weighted imaging; DWI, diffusion-weighted imaging; AP, arterial phase; PVP, portal venous phase; HBP, hepatobiliary phase; CR, complete response; SD, stable disease; mRECIST, modified Response Evaluation Criteria in Solid Tumors.

For patients with multiple lesions, we selected the largest lesion for both radiomic feature extraction and mRECIST evaluation. Tumor remission was assessed on the basis of the modified Response Evaluation Criteria in Solid Tumors (mRECIST) 3–4 months after TACE. The therapeutic response to TACE was classified into four grades: (1) complete response (CR), defined as complete disappearance of any intratumoral arterial enhancement in the targeted lesion; (2) partial response (PR), defined as a decrease of at least 30% in the sum of the diameters of viable target lesions (enhancement in the arterial phase); (3) progressive disease (PD), defined as an increase of at least 20% in the sum of the diameters of viable (enhancing) target lesions; and (4) stable disease (SD), defined as any case that did not qualify for PR or PD. According to mRECIST, patients with CR or PR were categorized as having an objective response (OR), whereas those with SD or PD were classified as having a nonobjective response (nOR) (**Figure 2**). Tumor response was independently assessed by two certified abdominal radiologists (J.Q. Ma and Y.Y. Liu) with 5 and 10 years of expertise in abdominal imaging on the basis of follow-up enhanced MRI or CT. Among the 160 enrolled patients, 112 were assigned to the OR group, and 48 were assigned to the nOR group. The patients were randomly divided into a training set (112 patients; OR = 78, nOR = 34) and a test set (48 patients; OR = 34, nOR = 14). In the external validation cohort, 32 patients were classified as OR and 6 as nOR.

Post-TACE follow-up was performed every 2–3 months with CECT/MRI, AFP, and liver function tests. OS (Overall survival) was defined as the time from the first TACE to death from study-related causes (as of September 1, 2024). Those alive or lost to follow-up by that date were censored.

### Construction of the radiomics model

**Figure 3:**
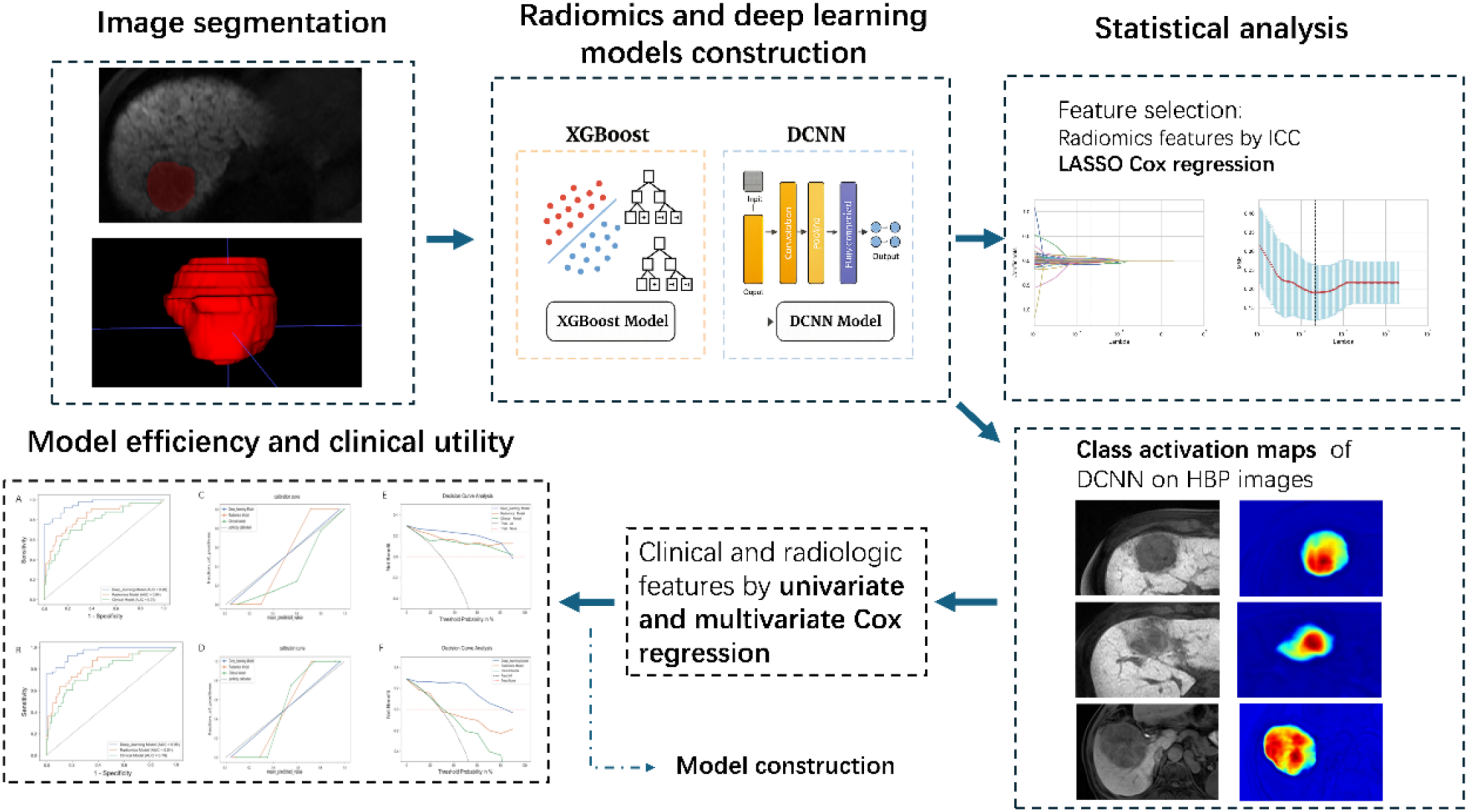
Model development and validation workflow. DCNN, deep convolutional neural network; ICC, intraclass correlation coefficient; HBP, hepatobiliary phase.

The model construction workflow is illustrated in **Figure 3**. HBP images from EOB-MRI were exported from the Picture Archiving and Communication System in Digital Imaging and Communications in Medicine format. The volume of interest of the tumor areas was manually delineated independently using ITK-SNAP version 3.8.0 (www.itk-snap.org) by two radiologists with 5 and 10 years of expertise in abdominal imaging. The intraclass correlation coefficient (ICC) was used to assess interobserver agreement, with an ICC value greater than 0.75 considered indicative of good reproducibility.

To enhance model stability and generalizability, all images were standardized and normalized to minimize interindividual variations in imaging parameters using min-max scaling. A harmonization step was used to correct for batch effect between scanners, ensuring consistency across different scanner models. High-throughput radiomic features were extracted from the preprocessed hepatobiliary phase images using the PyRadiomics module (version 3.0.1) in Python. A total of 1050 radiomics features were extracted for further analysis, including first-order statistical features, shape-based features, the gray level co-occurrence matrix (GLCM), the gray-level run-length matrix (GLRLM), the gray level zone matrix (GLSZM), the neighboring gray tone difference matrix (NGTDM), the gray level dependence matrix (GLDM) and wavelet features. These features were classified into three groups: geometric, intensity, and texture features. Texture features were extracted using four methods: GLCM, GLRLM, GLSZM, and NGTDM.

A total of 112 and 48 target lesions were included in the training cohort and test cohort, respectively. Feature selection was performed using the Mann–Whitney U test to identify significantly different radiomic features between response groups. Least absolute shrinkage and selection operator (LASSO) regression, combined with 10-fold cross-validation, was then used to select the features most relevant to the treatment response (**Supplementary Figure 1**). A final set of 26 radiomic features was retained. These features were linearly combined using their corresponding LASSO coefficients to calculate a radiomics score (Radscore) for each patient, which was implemented using the Python scikit-learn library (version 1.0.2). In addition, Shapley additive explanation (SHAP) value analysis was utilized to interpret the model and identify key features (**Supplementary Figure 1**).

### Construction of the deep learning model

To improve the stability of the model, all the input images had their pixel intensity ranges normalized to [0, 1]. Data augmentation techniques, including random rotation, horizontal and vertical flipping, and contrast adjustment, were applied to increase image diversity and reduce the risk of overfitting.

A deep convolutional neural network (DCNN) based on the ResNet50 architecture was used to construct a classification model for the HBP of EOB-MRI. ResNet50 employs a residual learning framework, which facilitates deeper network training and improved feature extraction. The fully connected layer was stabilized using batch normalization and regularized with a dropout layer (dropout rate = 0.5) to prevent overfitting. A Softmax activation function was used in the output layer to generate a probability distribution for binary classification.

Cross-entropy loss was adopted as the loss function to quantify the discrepancy between the predicted and actual class labels. Model parameters were optimized using the Adam optimizer combined with a cosine annealing learning rate decay schedule to promote efficient convergence and stable training. The dataset was randomly split into training (n = 112) and test (n = 48) sets at a 7:3 ratio. During training, both loss and accuracy were monitored in real time, and the model’s generalization performance was continuously evaluated using the test set. The model that performed best on the test set was retained.

### Nomogram construction

Clinical data preprocessing involved a stratified approach to handle missing values on the basis of variable type and missingness rate. Variables or samples with a missing data rate of 30% or higher were excluded to ensure data quality. For the remaining data, the distribution of continuous variables was assessed using the Shapiro–Wilk test. Normally distributed continuous variables were imputed using the mean, whereas nonnormally distributed variables were imputed using the median. Ordinal categorical variables were imputed with the median, and nominal categorical variables were imputed with the mode. Sensitivity analyses, including complete-case analysis and multiple imputation comparisons, were conducted to assess the robustness of the imputation.

After imputation, categorical variables were subsequently encoded using one-hot encoding. Continuous variables were standardized with Z score normalization if normally distributed or normalized with min–max scaling otherwise to reduce the influence of outliers. To address class imbalance, the synthetic minority oversampling technique (SMOTE) was applied to oversample the minority class and improve model generalizability.

Following data preprocessing, statistical analyses were performed. Clinical variables were categorized on the basis of standard reference ranges and initially analyzed using univariate analysis. Variables with statistical significance were then included, along with the Radscore, in a multivariate logistic regression model. A nomogram was constructed on the basis of the significant predictors identified through multivariate analysis. The final model was selected using a backward stepwise selection method, with the Akaike information criterion (AIC) used as the stopping rule. Stepwise logistic regression was used to calculate odds ratios (ORs) and 95% confidence intervals (CIs).

### Evaluation of different models

The predictive performance of different models was evaluated in terms of classification accuracy, sensitivity, specificity, and the area under the receiver operating characteristic curve (AUC) in both the training and test cohorts. Calibration curves were plotted to compare the predicted probabilities against the observed outcomes. Decision curve analysis (DCA) was performed to estimate the net clinical benefit over a range of threshold probabilities and evaluate the clinical utility of each model.

### Statistical analyses

All data processing and statistical analyses were conducted using SPSS (version 25.0) and Python (version 3.7.1). For continuous variables, the statistical tests included Student’s t test, Welch’s t test, and the Mann–Whitney U test, depending on the variance assumption. For categorical variables, the chi-square test or Fisher’s exact test was used. Statistical significance was defined as a two-sided p value < 0.05. The deep learning model was constructed using the PyTorch platform (version 1.13.1), whereas the radiomics model was built using an eXtreme gradient boosting (XGBoost) classifier. Tenfold cross-validation and ROC curve analysis were applied to validate the predictive performance of both models. Unless otherwise specified, all hyperparameters and configurations were set to the default values provided by the respective software platforms. In addition, a combined DLR model was constructed using independent factors selected from deep learning and radiomics features. Kaplan–Meier curves were used to assess the association between DLR scores and OS in the follow-up cohort. The Youden index determined the optimal threshold, dividing patients into high- and low-risk groups. Potential prognostic variables for OS were identified through univariate and multivariate Cox regression analyses, with statistical significance set at p < 0.05.

## Results

### Baseline characteristics of the patients

**Table 1** shows the results of univariate analysis comparing baseline demographic, clinical, and MRI characteristics between the training cohort (n = 112) and test cohort (n = 48). No statistically significant differences were observed between the two groups.

**Table 1:** Clinical characteristics of the training and validation sets.

Variables	Training set (n=112)	Validation set (n=48)	*p* value
**Age (years)**	58.2 [10.2]	58.6 [11.8]	1.000
**Sex**			0.818
Male	97 (86.6)	41 (85.4)	
Female	15 (13.4)	7 (14.6)	
**Hypertension**			0.653
No	55 (49.1)	39 (81.2)	
Yes	57 (50.9)	9 (18.8)	
**Diabetes**			0.411
No	93 (83.0)	43 (89.6)	
Yes	19 (17.0)	5 (10.4)	
Etiology			
HBV	105 (93.8)	46 (95.8)	
HCV	3 (2.7)	1 (2.1)	
HBV and HCV	1 (0.9)		
Others	3 (2.7)	1 (2.1)	
**Child–Pugh classification**			0.918
A	83 (74.1)	37 (77.1)	
B/C	29 (25.9)	11 (22.9)	
**BCLC stage**			0.890
A	60 (53.6)	27 (56.2)	
B	52 (46.4)	21 (43.8)	
**Treatment modality**			0.716
c-TACE	48 (42.9)	21 (43.8)	
DEB-TACE	20 (17.8)	8 (16.6)	
Combined TACE	44 (39.3)	19 (39.6)	
**AFP (ng/ml)**			0.470
≤400	87 (77.7)	34 (70.8)	
>400	25 (22.3)	14 (29.2)	
**PIVAK-II (mAU/ml)**			0.944
≤40	49 (43.8)	20 (41.7)	
>40	63 (56.2)	28 (58.3)	
**CEA (ng/ml)**			1.000
≤4.5	109 (97.3)	47 (97.9)	
>4.5	3 (2.7)	1 (2.1)	
**ALT (U/L)**			0.381
≤50	77 (68.8)	37 (77.1)	
>50	35 (31.2)	11 (22.9)	
**AST (U/L)**			0.469
≤40	60 (53.6)	22 (45.8)	
>40	52 (46.4)	26 (54.2)	
**GGT (U/L)**			0.730
≤60	54 (48.2)	21 (43.8)	
>60	58 (51.8)	27 (56.2)	
**Albumin (g/L)**			1.000
>40	23 (47.9)	52 (46.4)	
≤40	25 (52.1)	60 (53.6)	
**Total bilirubin (µmol/L)**			0.735
≤17.1	94 (83.9)	42 (87.5)	
>17.1	18 (16.1)	6 (12.5)	
**Prothrombin time (s)**			0.852
≤13	32 (66.7)	78 (69.6)	
>13	16 (33.3)	34 (30.4)	
**INR**			0.556
≤1.3	100 (89.3)	45 (93.8)	
>1.3	12 (10.7)	3 (6.2)	
**Radiological cirrhosis**			0.186
Present	34 (30.4)	9 (18.8)	
Absent	78 (69.6)	39 (81.2)	
**Tumor number**			0.825
<3	90 (80.4)	40 (83.3)	
≥3	22 (19.6)	8 (16.7)	
**Tumor size (mm)**	51.8 [25.6]	56.0 [27.2]	0.369
**Tumor margin**			0.147
Smooth margin	60 (53.6)	19 (39.6)	
Nonsmooth margin	52 (46.4)	29 (60.4)	
**Position**			0.204
Center	31 (27.7)	7 (14.6)	
Peripheral	71 (63.4)	36 (75.0)	
**Shape**			0.228
Round	62 (55.4)	24 (50.0)	
Irregular	34 (30.4)	19 (39.6)	
**Peritumoral hyperenhancement**			0.162
Yes	94 (83.9)	35 (72.9)	
No	18 (16.1)	13 (27.1)	
**HBP intensity**			0.734
Iso- to hyper	7(6.3)	1(2.1)	
hypo	89 (79.5)	40 (83.4)	
heterogenous	16 (14.3)	7 (14.6)	
**LR-M**			0.823
No	101 (90.2)	42 (87.5)	
Yes	11 (9.8)	6 (12.5)	

Data are presented as medians [interquartile ranges] or numbers (percentages). HBV, hepatitis B virus; HCV, hepatitis C virus; BCLC, Barcelona Clinic Liver Cancer; c-TACE, conventional transcatheter arterial chemoembolization; DEB-TACE, drug-eluting beads–transcatheter arterial chemoembolization; AFP, alpha-fetoprotein; PIVKA-II, protein induced by vitamin K absence or antagonist-II; CEA, carcinoembryonic antigen; ALT, alanine amino-transferase; AST, aspartate amino-transferase; GGT, γ-glutamyl transpeptidase; HBP, hepatobiliary phase; LR-M, LI-RADS M (definite or probable malignancy, not specific for hepatocellular carcinoma).

Bold values indicate variable headings and subgroup labels in the table.

**Table 2** summarizes the univariate and multivariate analyses comparing baseline characteristics between the OR and nOR groups. In the univariate analysis, significant differences were found in BCLC stage (P = 0.023), γ-glutamyl transpeptidase (GGT, P = 0.038), tumor number (P = 0.001), tumor size (P = 0.001), and tumor location (P = 0.012). Multivariate analysis identified BCLC stage (P = 0.035), tumor number (P = 0.015), and tumor size (P = 0.013) as independent predictors of treatment response.

**Table 2:** Univariate and multivariate analyses of the baseline characteristics of the patients in the OR and nOR groups.

Variables	Univariate analysis			Multivariate analysis	
	OR (n=112)	nOR (n=48)	*p* value	OR (95% CI)	*p* value
**Age (years)**	58.2 [10.2]	58.5 [11.8]	0.51		
**Sex**			0.152		
Male	94 (83.9)	44 (91.7)			
Female	18 (16.1)	4 (8.3)			
**Child–Pugh classification**			0.834		
A	83 (74.1)	37 (77.1)			
B/C	29 (25.9)	11 (22.9)			
**BCLC stage**			0.023*	0.08 (-0.09,0.26)	0.035*
A	64 (57.1)	23 (47.9)			
B	48 (42.9)	25 (52.1)			
**Treatment modality**			0.435		
c-TACE	48 (42.9)	21 (43.8)			
DEB-TACE	17 (15.2)	11 (22.9)			
Combined TACE	47 (42.0)	16 (33.3)			
**AFP (ng/ml)**			0.08		
≤400	87 (77.7)	34 (70.8)			
>400	25 (22.3)	14 (29.2)			
**GGT (U/L)**			0.038*	0.03 (-0.13~0.18)	0.754
≤60	59 (52.7)	16 (33.3)			
>60	53 (47.3)	32 (66.7)			
**Tumor number**			0.001*	0.16 (-0.06~0.38)	0.015*
<3	97 (86.6)	33 (68.8)			
≥3	15 (13.4)	15 (31.2)			
**Tumor size (mm)**	48.5 [19.8]	63.9 [34.7]	0.001*	0 (0~0.01)	0.013*
**Position**			0.012*	0.01 (-0.14~0.16)	0.881
Center	27 (24.1)	11 (22.9)			
Peripheral	82 (73.2)	25 (52.1)			
Others	3 (2.7)	12 (25.0)			

* p<0.05; the data are presented as medians [interquartile ranges] or numbers (percentages). OR, objective response; nOR, nonobjective response; CI: confidence interval; BCLC, Barcelona Clinic Liver Cancer; c-TACE, conventional transcatheter arterial chemoembolization; DEB-TACE, drug-eluting beads transcatheter arterial chemoembolization; AFP, alpha-fetoprotein; GGT, γ-glutamyl transpeptidase.

Bold values indicate variable headings and subgroup labels in the table.

### Interobserver reproducibility of feature extraction

There was no significant discrepancy between the measurements of the two radiologists for each selected feature (p>0.05). The interobserver agreement between the two radiologists ranged from 0.87 to 1.00. These findings indicated robust reproducibility of feature extraction between observers. Finally, all outcomes were derived from the measurements of radiologist A.

### Nomogram construction

**Figure 4:**
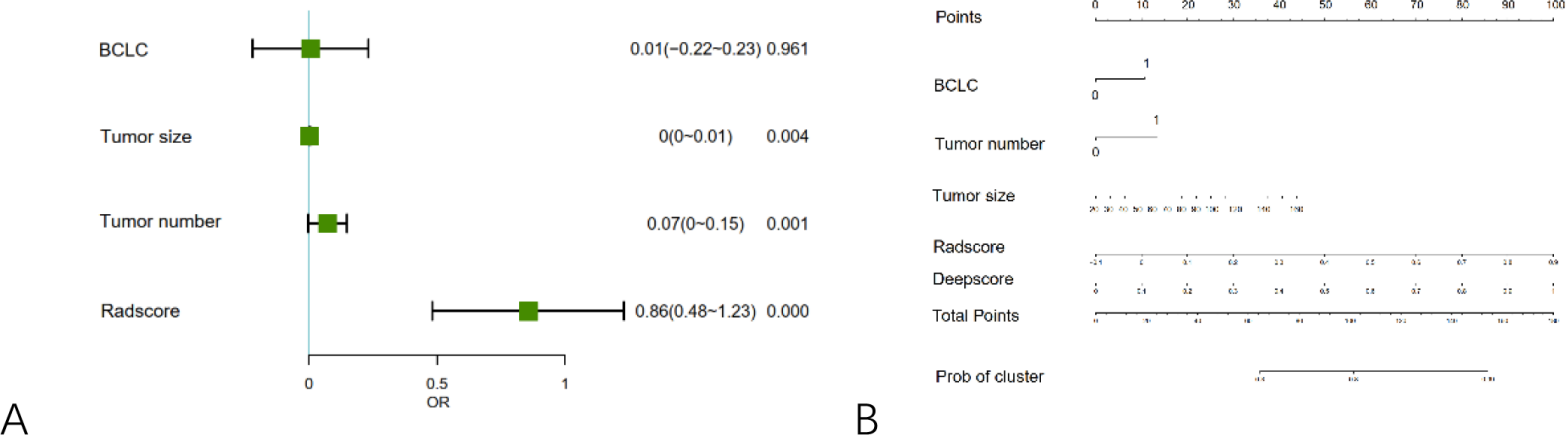
Results of multivariate logistic regression and nomogram construction. **(A)** Forest plot of significant predictors identified in multivariate analysis.; **(B)** Nomogram incorporating clinical, radiomic, and deep learning features for predicting treatment response.

On the basis of multivariate regression analysis, three independent clinical predictors (BCLC stage, tumor number, and tumor size) along with the Radscore were included in the final model (**Figure 4a**). A nomogram combining radiomics and deep learning features was constructed to predict treatment response to initial TACE (**Figure 4b**).

### Evaluation of the clinical efficacy of the three models

**Figure 5:**
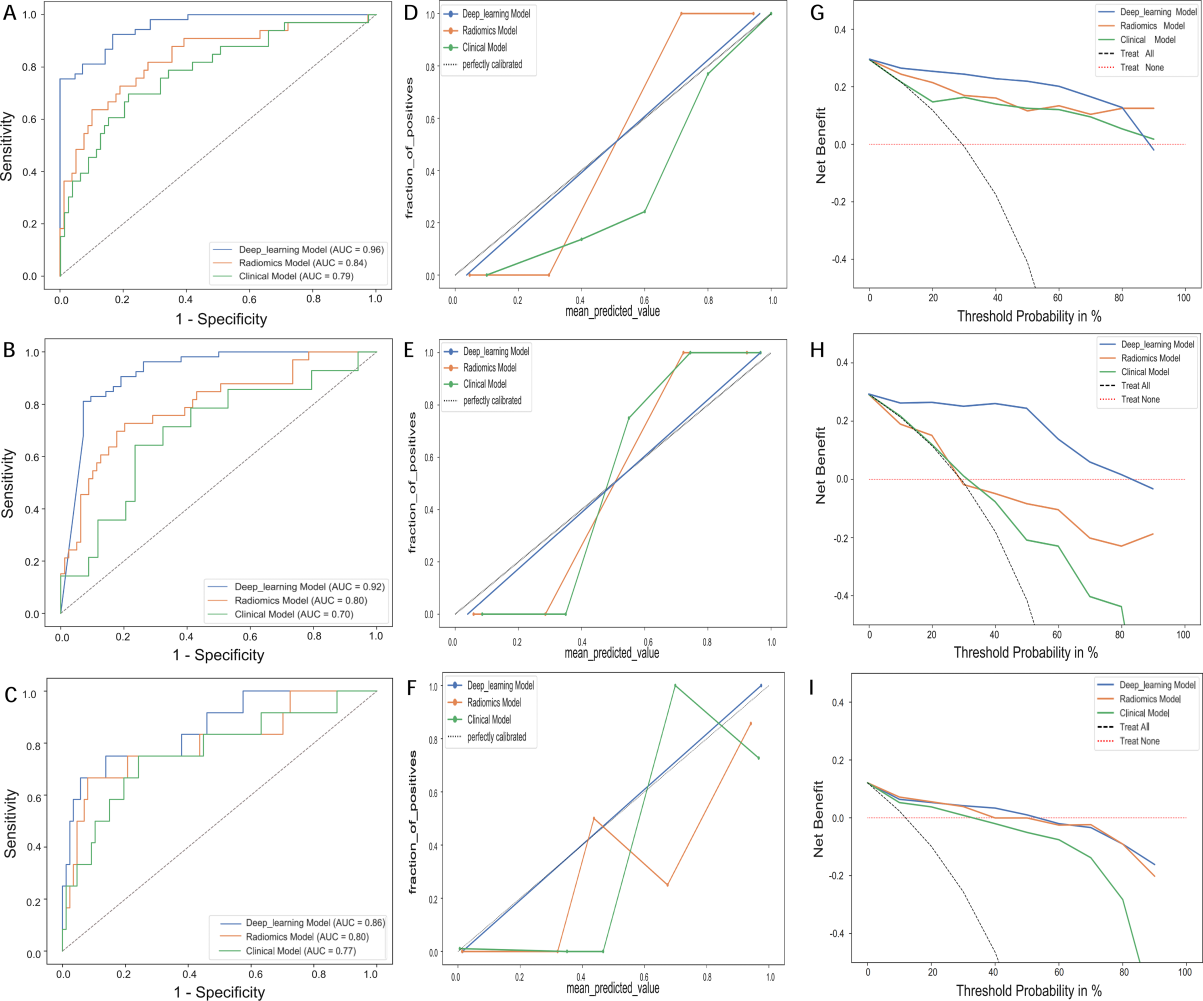
Evaluation of model performance in the training, test and external validation cohorts. A-C: Receiver operating characteristic curves (ROC) of the clinical, radiomics and deep learning models in **(A)** training, **(B)** test and **(C)** external validation sets for predicting TACE response. **(D-F)** Calibration curves of Clinical model, Radiomics model and Deep learning model for predicting TACE response in **(D)** training, **(E)** test and **(F)** external validation sets. **(G-I)** Decision curve analysis (DCA) for the three models in the **(G)** training, **(H)** test and **(I)** external validation sets. TACE, transarterial chemoembolization.

Prediction models were developed using extracted radiomic features based on XGBoost and DCNN (**Figure 3**). **Table 3** summarizes the performance of the clinical, XGBoost, and DCNN models, as evaluated by the AUC, accuracy, sensitivity, and specificity. In the training cohort, the clinical, XGBoost, and DCNN models achieved accuracies of 0.72, 0.85, and 0.90; sensitivities of 0.79, 0.85, and 0.87; and specificities of 0.77, 0.85, and 0.93, respectively. In the test cohort, the accuracies were 0.75, 0.85, and 0.94; the sensitivities were 0.76, 0.83, and 0.84; and the specificities were 0.71, 0.79, and 0.87 for the clinical, XGBoost, and DCNN models, respectively. In the external validation cohort, the accuracies were 0.78, 0.81, and 0.88; the sensitivities were 0.75, 0.91, and 0.87; and the specificities were 0.85, 0.73, and 0.81 for the clinical, XGBoost, and DCNN models, respectively. In terms of the AUC, the DCNN model significantly outperformed both the clinical and XGBoost models in the training set (AUC = 0.96, 95% CI: 0.93–0.99; P < 0.05) compared with the clinical model (AUC = 0.79, 95% CI: 0.73–0.85) and the XGBoost model (AUC = 0.84, 95% CI: 0.78–0.90). This superior performance was maintained in the test set and the external validation set, where the DCNN model achieved AUCs of 0.92 and 0.86, outperforming the clinical model (AUCs = 0.70 and 0.77) and the XGBoost model (AUCs = 0.80 and 0.80), as shown in **Figures 5A-C**.

**Table 3:** Predictive performance of various models in the training, test and external validation sets.

Classifiers	AUC	Accuracy	Sensitivity	Specificity
Training set				
Clinic	0.79 (0.73, 0.85)	0.72	0.79	0.77
Radiomics	0.84 (0.78, 0.90)	0.85	0.85	0.85
Deep learning	0.96 (0.93, 0.99)	0.90	0.87	0.93
Test set				
Clinic	0.70 (0.63, 0.77)	0.75	0.76	0.71
Radiomics	0.80 (0.74, 0.86)	0.85	0.83	0.79
Deep learning	0.92 (0.88, 0.96)	0.94	0.84	0.87
External validation set				
Clinic	0.77 (0.70, 0.84)	0.78	0.75	0.85
Radiomics	0.80 (0.73, 0.87)	0.81	0.81	0.73
Deep learning	0.86 (0.81, 0.91)	0.88	0.87	0.91

AUC, area under the curve; CI, confidence interval.

Across the training, test and external validation cohorts, the models exhibited a stepwise improvement in predictive performance, with the XGBoost model outperforming the clinical model and the DCNN model demonstrating the highest overall accuracy. These findings indicate strong generalizability for predicting TACE response in HCC patients. The calibration curves showed excellent agreement between the predicted probabilities and observed outcomes for the DCNN model, underscoring its reliability and predictive accuracy (**Figures 5D-F**). Furthermore, decision curve analysis demonstrated that the DCNN model yielded the greatest net clinical benefit compared with both the clinical and XGBoost models, supporting its potential utility in clinical decision-making (**Figures 5G-I**).

### Evaluation of survival

**Figure 6:**
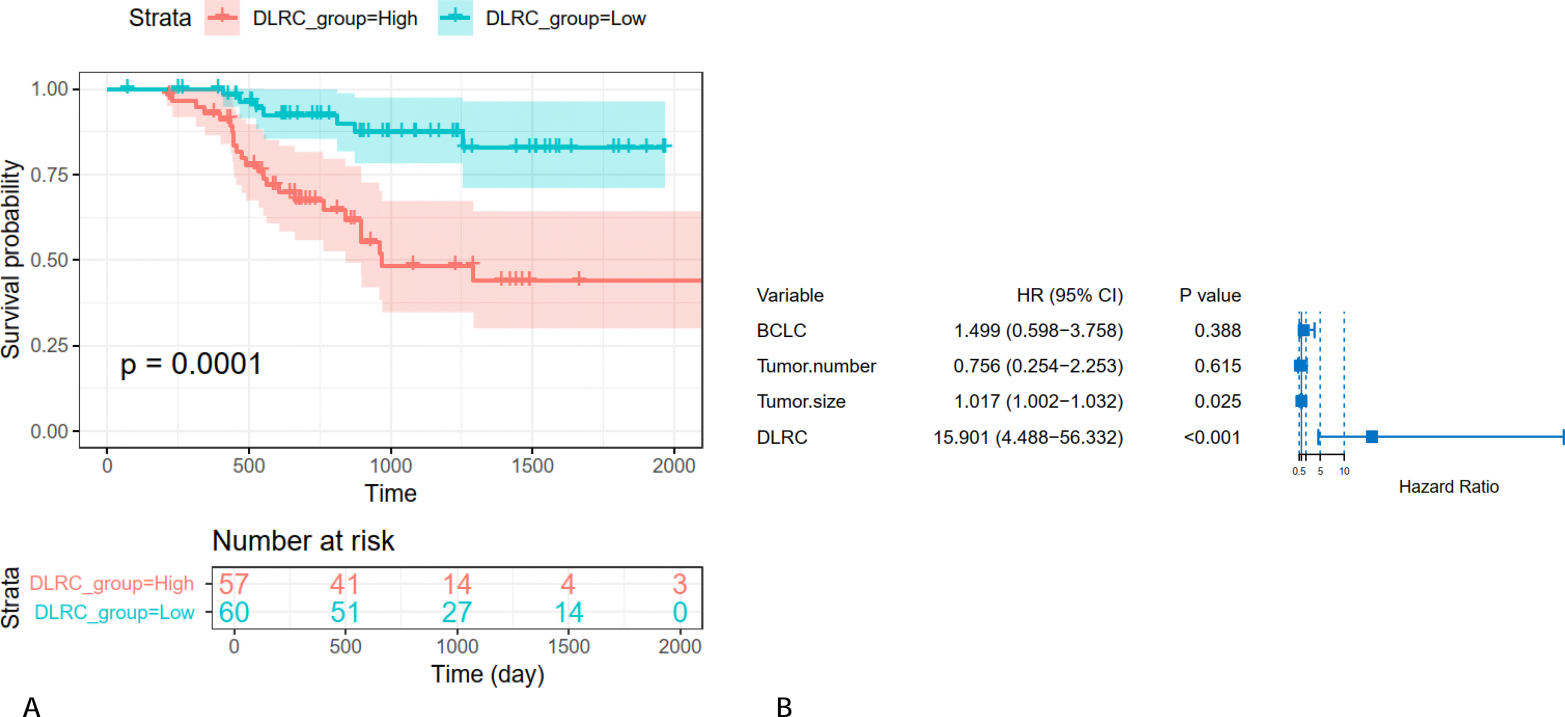
Kaplan-Meier curves of OS and forest plot for HCC survival. **(A)** Kaplan–Meier analysis of OS stratified by DLR-based high- and low-risk groups; **(B)** Forest plot illustrating multivariate Cox regression results for OS. DLR, deep learning and radiomics; OS, overall survival.

117 patients completed follow-up for OS and were subsequently assessed to determine the prognostic value of the DLR model. Kaplan-Meier analysis revealed that patients with higher DLR scores had significantly shorter OS (log-rank test, p = 0.0001) (**Figure 6A**). Moreover, the DLR model remained as an independent predictor of OS in the multivariate Cox regression analysis (HR: 15.9, 95% CI: 4.49-56.33; p < 0.001). Tumor diameter also showed statistical significance in the multivariate Cox analysis (**Figure 6B**).

## Discussion

This study developed and validated radiomic and deep learning models based on HBP of EOB-MRI for predicting the treatment response of patients with HCC (>3 cm) to TACE. The results demonstrated that the radiomics model and deep learning model based on HBP both achieved excellent predictive performance (AUC = 0.84 and 0.96, respectively) for preoperative prediction in HCC patients prior to TACE, outperforming the clinical model (AUC = 0.79). Moreover, the two models demonstrated good stability in the test and external validation sets, with AUCs of 0.80 and 0.80 for the radiomics model and 0.92 and 0.86 for the deep learning model, respectively. In addition, DLR model output was identified as an independent prognostic factor for overall survival (hazard ratio: 15.9, 95% CI: 4.49-56.33; p < 0.001).

Multiple studies have shown that MRI-based radiomics and deep learning models can effectively predict treatment response to TACE (13, 15, 17–20, 22, 23, 26, 31). Kong et al. (17) developed a radiomics model based on a single T2-weighted imaging (T2WI) sequence. Using LASSO regression, six radiomics features were selected to calculate a Radscore, achieving AUCs of 0.812 and 0.866 in the training and validation datasets, respectively, which is notable for a single-sequence mode. Similarly, Kuang et al. (19) reported comparable performance between T2WI-based and arterial phase (AP)-based models, with AUCs of 0.81 and 0.82, respectively, in a multicenter cohort. However, the predictive performance of single-sequence models has shown considerable variation across different studies and patient cohorts (20, 22, 32). The reported AUCs for T2WI-based models range from 0.602 to 0.840 (13, 17–20, 22), with similar variability observed for AP-based models (AUCs: 0.779-0.89) (19, 22, 32). Zhao et al. (31) reported that models constructed using the portal venous phase (PVP) outperformed those based on the AP and delayed phase (DP). In our study, the HBP-based model achieved robust predictive performance, with AUCs of 0.84 in the training set and 0.80 in the test set, indicating strong accuracy and stability. Despite these variations, there is general agreement that multiparametric MRI models achieve superior predictive power (AUCs: 0.791-0.941), as they provide more biological information on tumor heterogeneity (14, 18, 20, 23, 31, 33). Moreover, including peritumoral regions can further enhance model performance. Zhao et al. (23) demonstrated that incorporating a 3 mm peritumoral margin with the intratumoral region improved performance, achieving AUCs of 0.884 and 0.911 in the training and validation cohorts, respectively.

Overall, deep learning models have shown greater robustness and generalizability. Chen et al. (15) constructed both radiomics and deep learning models on the basis of four MRI sequences (T2WI, AP, PVP, and DP), achieving the highest reported AUCs to date: 0.941 using LASSO and 0.927 using a deep neural network (DNN). However, the radiomics model exhibited a substantial decline in the validation set, suggesting potential overfitting, whereas the DNN model maintained consistent performance. Similarly, Svecic et al. (34) proposed a novel framework, the spatial–temporal discriminant graph neural network, which achieved an accuracy of 0.932 in cross-validation using multiphase DCE-MRI. Nevertheless, to date, no studies have specifically evaluated models based solely on the HBP or systematically assessed its added predictive value.

The clinical model is simple and accessible but offers lower predictive performance. In contrast, the radiomics model improves accuracy and sensitivity, though it still lags behind deep learning, particularly in specificity. The deep learning model excels in all metrics, providing superior performance, but requires large datasets, high computational resources, and is harder to interpret. Moreover, the deep learning and radiomics models enabled not only short-term treatment response prediction but also prognostic risk stratification through the integrated DLR model. By combining independent deep learning and radiomics features into a composite DLR score, patients were classified into high- and low-risk groups, with the low-risk group showing significantly longer overall survival (p < 0.001). In multivariate Cox regression, both the DLR score and tumor diameter were identified as independent prognostic factors for overall survival.

Previous studies have explored imaging-based models for predicting TACE outcomes. Sun et al. (35) developed a deep learning–radiomics–clinical model integrating arterial-phase contrast-enhanced CT features and clinical variables to predict treatment response and survival in intermediate-stage HCC. Liu et al. (20) constructed a two-center MRI radiomics–clinical model based on T2-weighted imaging that accurately predicted post-TACE response and prognosis, outperforming models using clinical or imaging features alone. Lee et al. (11) reported certain EOB-MRI features, including tumor multiplicity and marginal irregularity, were associated with poor outcomes after cTACE in small HCCs. Although EOB-MRI has proven valuable for noninvasive pathological and prognostic evaluation (36, 37), few studies have applied it for machine learning–based prediction. Cannella et al. (26) built an HBP radiomics model in 51 small HCCs (<3 cm) and achieved moderate performance (AUC = 0.790), with a sensitivity of 58.8%, and a specificity of 90.1%. In comparison, our HBP-based DCNN model achieved higher predictive accuracy (AUC = 0.96, sensitivity = 0.87, specificity = 0.93) in a larger cohort including BCLC stage A and B patients. Furthermore, the integrated DLR model provided independent prognostic value for overall survival, demonstrating that HBP-based machine learning can serve as a simplified yet effective approach for both treatment response prediction and prognostic assessment.

The HBP of EOB-MRI is effective in evaluating treatment response due to its ability to assess not only the morphological features of the tumor but also the functional aspects of the liver tumor. Compared to other sequences, the HBP provides a clearer visualization of the tumor’s shape, size, and margins, which are highly valuable in predicting CR or OR after TACE. Furthermore, the degree of contrast uptake in the HBP is influenced by the tumor’s differentiation and microvascular characteristics. Since TACE primarily targets tumor vasculature, areas with poor contrast uptake in the HBP may indicate incomplete response or tumor recurrence due to residual or refractory tumor cells with abnormal microvascular structures. Thus, HBP can effectively highlight regions that may require further intervention or additional monitoring. However, the underlying mechanisms still require further investigation.

This study confirmed that several previously reported clinical factors, including BCLC stage, tumor number, and tumor diameter, are significantly associated with the treatment response to TACE. The BCLC stage and its subclassifications significantly affect response rates, which is consistent with prior studies emphasizing the value of BCLC staging in both prognostic assessment and treatment stratification in patients with HCC (14). The tumor number is also an independent predictor, with patients presenting with multiple lesions being more likely to exhibit a poor response. Additionally, a larger tumor diameter was also associated with a decreased likelihood of response. These findings align with existing evidence suggesting that a greater tumor burden, including tumor number and diameter, may impair treatment efficacy (4). Larger and multiple tumors, often accompanied by intratumoral heterogeneity or satellite lesions, pose a greater challenge to complete embolization and a sustained response. Consequently, these significant clinical predictors were incorporated into the nomograms alongside radiomics and deep learning features, enhancing the comprehensive predictive capability of the integrated models.

In contrast, tumor location and the AFP level were not found to be independent predictors in our model, despite previous studies highlighting their prognostic relevance (14, 15). The discrepancy regarding tumor location may be partly attributed to methodological differences. While prior research often categorizes tumor location by anatomical lobe (left, right, or bilobar) or specific segment (e.g., the caudate lobe), our study classified lesions as either central or peripheral, similar to Asano et al. (12). The negative result may be due to the presence of massive tumors (>10 cm), many of which are bilobar, which may have confounded the assessment of location as an isolated factor. Additionally, while AFP has been validated as an important prognostic biomarker in HCC and is frequently used to assess long-term survival outcomes in patients undergoing TACE (14), its utility in predicting treatment response remains inconclusive. Our study applied a threshold of 400 ng/mL, whereas other studies have used lower cutoffs (e.g., 20 or 200 ng/mL), potentially affecting comparative outcomes (14). Moreover, the imbalanced number of patients in the OR and nOR groups may introduce clinical heterogeneity, further influencing the predictive power of AFP and other variables.

Our study also assessed whether radiologic features, including tumor shape, margin, peritumoral hyperenhancement, HBP intensity and LR-M, were associated with tumor response. These features are significantly related to pathological factors such as MVI and poor tumor differentiation, both of which contribute to a poor prognosis. However, no significant associations were found. These findings suggest that these prognostic indicators may influence the prognosis in other ways, such as undetected residual lesions on imaging, a higher recurrence rate or varied sensitivity to multiple rounds of TACE. Interestingly, Yoo et al. (38) reported a case of viable HCC foci within a radiologically complete response lesion after TACE in a patient with pathologically confirmed thickened trabeculae-type HCC. These findings highlight the limitations of the naked eye assessment of radiologists in capturing subtle imaging details and emphasize the value of machine learning and deep learning approaches.

Our study has several limitations. First, this was a retrospective study in a single center with a relatively small population, and the external sample size was also limited. Second, most participants had HBV-related HCC, which may limit generalizability to Western populations, where alcoholic and metabolic-related liver diseases are more common. Third, the types of TACE procedures used varied and included cTACE, DEB-TACE, and combination TACE. Although no significant differences in efficacy were observed, procedural variability may still have influenced the outcomes. Fourth, treatment response was assessed using only the HBP sequence, whereas multiparameter MRI is more often recommended and more consistent with standard clinical protocols. Additionally, our study focused solely on radiomic and deep learning features from the HBP sequence for predicting TACE efficacy, without exploring the underlying biological mechanisms or interpretability of these models. Future research should focus on validating robust predictive models using multisequence EOB-MRI in large, prospective, multicenter cohorts.

## Conclusion

In conclusion, radiomics and deep learning model based on HBP of EOB-MRI were developed and validated for the noninvasive preoperative prediction of treatment response in patients with HCC undergoing TACE. The models demonstrated strong predictive performance and may serve as a valuable tool to assist clinicians in identifying patients most likely to benefit from TACE and guiding individualized treatment strategies.

## Data Availability

The original contributions presented in the study are included in the article/**Supplementary Material**. Further inquiries can be directed to the corresponding author.

## Glossary

HCC: Hepatocellular carcinoma
TACE: Transarterial chemoembolization
BCLC: Barcelona Clinic Liver Cancer
EOB-MRI: Gadoxetic acid–enhanced magnetic resonance imaging
Gd-EOB-DTPA: Gadoxetic acid
HBP: Hepatobiliary phase
MRI: Magnetic resonance imaging
cTACE: Conventional transarterial chemoembolization
DEB-TACE: Drug-eluting bead–transarterial chemoembolization
AFP: Alpha-fetoprotein
VOI: Volume of interest
ICC: Intraclass correlation coefficient
GLCM: Gray-level co-occurrence matrix
GLRLM: Gray-level run length matrix
GLSZM: Gray-level size zone matrix
NGTDM: Neighboring gray tone difference matrix
GLDM: Gray-level dependence matrix
LASSO: Least absolute shrinkage and selection operator
SHAP: Shapley Additive Explanation
DCNN: Deep convolutional neural network
AUC: Area under the receiver operating characteristic curve
DCA: Decision curve analysis
OR: Objective response
nOR: Nonobjective response
CI: Confidence interval
AI: Artificial intelligence
DNN: Deep neural network
DLR: Deep learning and radiomics
