# Supplementary material for: AI models based on gadoxetic acid–enhanced MRI to predict treatment response and prognosis after TACE in hepatocellular carcinoma
Source: Front Oncol. 2026 Apr 28;16:1738531. doi: 10.3389/fonc.2026.1738531 (PMC13160757; doi:10.3389/fonc.2026.1738531)
Supplement: Supplementary file 1 [file DataSheet1.docx]

a
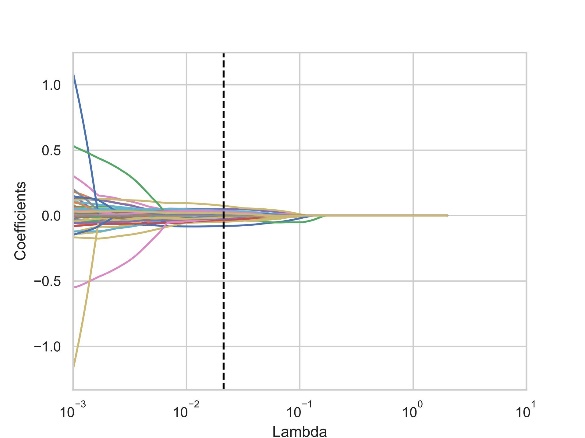
 b
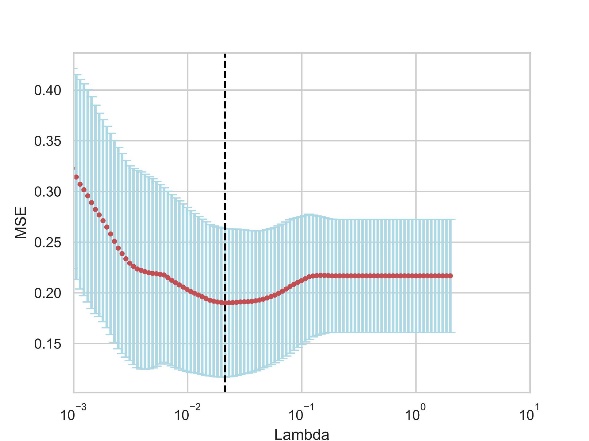


c
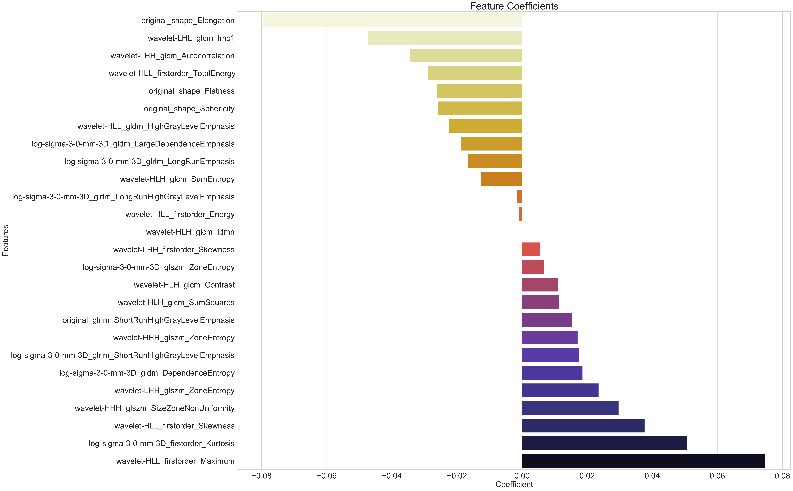


d
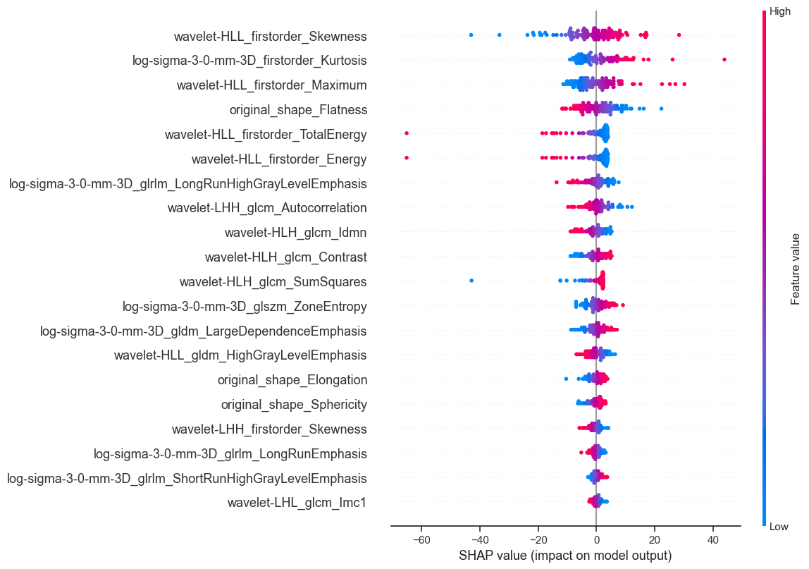


Supplementary Figure S1. Radiomic feature selection and model interpretation. (a) LASSO coefficient profiles of 26 radiomic features.; (b) Selection of the optimal tuning parameter (λ) in the LASSO model via 10-fold cross-validation using the minimum criteria. The vertical dotted line indicates the optimal value of λ, which resulted in five features with nonzero coefficients; (c) Bar chart displaying the contribution of individual radiomic features to the radiomics score (Radscore), based on their respective model coefficients. LASSO, least absolute shrinkage and selection operator. (d) SHAP summary plot showing the impact of individual radiomic features on the model output. LASSO, least absolute shrinkage and selection operator.
