# Supplementary material for: AI models based on gadoxetic acid–enhanced MRI to predict treatment response and prognosis after TACE in hepatocellular carcinoma
Source: Front Oncol. 2026 Apr 28;16:1738531. doi: 10.3389/fonc.2026.1738531 (PMC13160757; doi:10.3389/fonc.2026.1738531)
Supplement: Supplementary file 2 [file Table1.docx]

**Supplementary Table 1. MR Imaging Acquisition of hepatobiliary phase**

| **MR scanners** | **Telsa** | **Parameters** | | | | | | |
| --- | --- | --- | --- | --- | --- | --- | --- | --- |
|  |  | **TR (ms)** | **TE (ms)** | **FOV (mm)** | **Matrix** | **Reverse angle (°)** | **Band width** | **Thickness (mm)** |
| Prisma, Siemens Healthcare | 3.0 T | 5.01 | 2.3 | 380×305 | 135×224 | 15 | 558 | 0.8 |
| Signa HDxt, GE Healthcare | 1.5 T | 3.37 | 1.33 | 400×320 | 320×192 | 15 | 83.3 | 5 |

Note: TR, repetition time; TE, echo time; FOV, field of view.
